# TNFSF14‐HVEM/LTβR Exacerbates Keratinocyte Abnormalities and IMQ‐Induced Psoriatic Skin Inflammation via Activating NF‐κB/TWIST1 Signalling Pathway

**DOI:** 10.1111/jcmm.70774

**Published:** 2025-08-06

**Authors:** Sheng‐jie Long, Quan‐you Zheng, Feng Xu, Gui‐qing Li, Jian Chen, Wen‐jie Chen, Jiang‐mei Xu, Xiao‐lin Gao, Shen‐ju Liang, Gui‐lian Xu

**Affiliations:** ^1^ Department of Immunology, Basic Medicine College Army Medical University Chongqing China; ^2^ Urinary Nephropathy Center The Second Affiliated Hospital of Chongqing Medical University Chongqing China; ^3^ Department of Urology, 958th Hospital, Southwest Hospital Army Medical University Chongqing China; ^4^ Department of Nephrology, Southwest Hospital Army Medical University Chongqing China; ^5^ Deprtment of Dermatology, Xinqiao Hospital Army Medical University Chongqing China; ^6^ Department of Nephrology The Ninth People's Hospital of Chongqing Chongqing China; ^7^ Department of Rheumatism and Immunology, Daping Hospital Army Medical University Chongqing China

**Keywords:** keratinocytes abnormalities, NF‐κB/TWIST1, psoriasis, TNFSF14‐HVEM/LTβR

## Abstract

Psoriasis (PS) is a chronic autoimmune skin disease that poses a serious threat to over 100 million patients worldwide. An increasing number of studies have indicated that keratinocytes (KCs) play an essential role in the inflammatory progression of PS. The present study found that tumour necrosis factor superfamily member 14 (TNFSF14) and its two receptors were up‐regulated in IMQ‐primed KCs and psoriatic skin sections. It also revealed that blocking TNFSF14 signalling (via gene knockout or injections) with its receptors' soluble fusion proteins lymphotoxin beta receptor (LTβR)–immunoglobulin G Fc domain (LTβR‐IgGFc) and herpesvirus entry mediator–IgGFc (HVEM‐IgGFc) significantly alleviated imiquimod (IMQ)‐induced psoriatic skin inflammation by attenuating epidermal hyperplasia, decreasing cellular proliferation, keratinisation, apoptosis, and inflammatory response. Accordingly, direct stimulation with recombinant TNFSF14 markedly enhanced KC abnormalities as evidenced by aggravated cell proliferation and keratinisation, increased cellular apoptosis, and up‐regulated inflammatory cytokine expression. Mechanistic studies demonstrated that TNFSF14 mediates KC abnormalities via the nuclear factor‐kappa B (NF‐κB)/TWIST1 pathway. Taken together, our study findings indicate that TNFSF14‐HVEM/LTβR promotes KC dysfunction and IMQ‐induced psoriatic skin inflammation via enhancing NF‐κB/TWIST1 signalling and suggest that TNFSF14 is a promising therapeutic strategy for the clinical treatment of PS.

## 1 Introduction

PS, a prevalent chronic and recurrent autoimmune skin disease characterised by sustained inflammation, disordered proliferation and differentiation of KCs, and uncontrolled angiogenesis, affects 2%–3% of the population worldwide [1]. There are several forms of PS, including vulgaris (most common), erythrodermic, guttate, pustular, plaque, and inverse [1]. PS is always accompanied by other conditions, such as psoriatic arthritis, cardiovascular disease and diabetes, causing physical and mental distress for over 100 million individuals and posing global health issues [2, 3]. It is widely believed that multiple factors, including environmental and genetic, promote the progression of PS, but its precise aetiology is complex and remains poorly understood [4, 5].

Mounting evidence has revealed that the crosstalk between excessive skin immune cells and disordered KC activation contributes to PS progression. The abnormal interactions between KCs and interleukin (IL)‐17‐producing T cells are responsible for the initiation and maintenance of PS [6]. KCs, the major components of the epidermal layer, are considered an essential element of the innate immune system and play a critical role in regulating the adaptive immune response [7]. Moreover, increasing research suggests that KCs are not only the executors of immune cell function in the PS IL‐23/IL‐17 axis but are also trigger factors [8]. Pasparakis et al. observed spontaneous PS‐like plaques on the dorsal skin of neonate mice upon specific knockout of IκB kinase (Ikk2) in KCs [9]. Ikk2 deficiency in KCs leads to the dramatic overexpression of cytokines and chemokines in a TNFR1/IL‐24/STAT3‐dependent manner [9]. Additionally, the inducible deletion of AP‐1 transcription factor subunit JunB and c‐Jun in KCs showed a similar PS‐like phenotype [10]. Moreover, recombination activating gene 2 (Rag2) protein‐deficient JunB/c‐Jun^−/−^ mice (T and B cell defects) still exhibited epidermal thickening, parakeratosis, vasodilation, and micro‐abscesses, indicating a minor role of T and B cells in the pathology of PS [10]. Thus, it is believed that KCs are an essential instigator of skin inflammation in PS. Although the underlying mechanism remains unclear, accumulating evidence suggests that the interaction between “inflammation–inflammation” [11, 12, 13] and “inflammation–abnormal biological function” [14, 15, 16, 17] in KCs is crucial to the pathogenesis of PS. TNFSF14, a key regulator of inflammation, provides co‐stimulation signals and helps maintain immune homeostasis by binding to herpesvirus entry mediator–IgGFc (HVEM) and lymphotoxin beta receptor (LTβR) [18, 19, 20, 21]. Interestingly, TNFSF14 signalling has different effects under specific conditions [22, 23]. In recent years, TNFSF14 has attracted more attention as a potential enhancer in some skin disorders. Rana et al. discovered that the TNFSF14‐HVEM pathway specifically controlled KC hyperplasia, periosteal protein expression, and inflammatory cytokine release in a murine model of atopic dermatitis [24]. In addition, blocking TNFSF14 or HVEM/LTβR attenuated collagen deposition and thickening in mice with bleomycin‐induced skin fibrosis [25]. As to PS, Gupta et al. confirmed that mice devoid of TNFSF14, or deletion of either of its receptors in KCs, were protected from developing IMQ‐induced psoriatic [26]. Further single cell RNA‐seq analysis of PS patient biopsies, LTβR and HVEM transcripts were found strongly expressed in KCs, and TNFSF14 was upregulated in T cells [26]. Moreover, in vitro, TNFSF14 stimulation upregulated a broad spectrum of genes in human KCs that are clinical features of PS skin lesions [26], suggesting a closely correlation of TNFSF14‐HVEM/LTβR pathway with PS development. However, the effects and underlying mechanism of TNFSF14 on PS pathology and KC dysfunction remain unclear.

Here we explored the role of TNFSF14 in the pathogenesis of PS by focusing on its biological effects on KC abnormalities, including proliferation, keratinisation, apoptosis, and inflammation, by using a murine model of IMQ‐induced psoriatic skin inflammation and IMQ‐treated human KCs [27].

## 2 Materials and Methods

### 2.1 Human Skin Tissue

Skin tissue sections of healthy volunteers and patients with PS were obtained from the Department of Dermatology at Chongqing 958 Hospital. Patients had no autoimmune diseases or chronic inflammation, and had not used immunosuppressants, glucocorticoids, biologics or similar drugs at least 1 month before participating in the trial.

### 2.2 Mice and Treatment

TNFSF14 knockout mice (TNFSF14^−/−^, C57BL/6) were kindly provided by Dr. Pfeffer (University of Düsseldorf, Germany), whereas wild‐type mice (TNFSF14^+/+^, C57BL/6) were purchased from Huafukang Biotechnology Co. Ltd. (Beijing, China). The mice were housed at constant temperature and humidity in a specific pathogen‐free facility; female mice (8–12 weeks of age; 22–24 g each) were used in all experiments. The animal experiment was approved by the Institutional Animal Care and Use Committee of Army Medical University. To induce PS‐like skin inflammation in mice, 1 day prior to the experiments, the dorsal skin was carefully shaved (2.0 × 3.0 cm) to avoid trauma, and the exposed area was topically treated with 62.5% IMQ cream (Med‐Shine Pharmaceutical, Sichuan, China) for 5 consecutive days. The mice in the control group were treated with an equal dose of Vaseline cream. In the blocking experiments, TNFSF14^+/+^ mice were randomly divided into control human IgGFc (Sino Biological, Beijing, China) and herpesvirus entry mediator–immunoglobulin G Fc domain (HVEM‐IgGFc; Sino Biological, Beijing, China) plus LTβR‐IgGFc (murine extramembrane LTβR fused with human IgGFc fragment; Zoonbio, Nanjing, China) groups. The mice received a peritoneal injection of control human IgGFc (100 μg) or HVEM‐IgGFc (50 μg) combined with LTβR‐IgGFc (50 μg) 2 h before with 50 mg/day of 5% IMQ treatment on Days 1, 3, and 5.

### 2.3 Cells and Treatment

Human immortalised KCs (HaCaT) were provided by American Type Culture Collection (Virginia, USA) and cultured in complete medium (minimum essential medium [MEM] + 10% fetal bovine serum [FBS] + 1% penicillin–streptomycin) at 37°C and 5% CO2. Single‐cell suspensions were seeded on a six‐well plate. Once the cells covered approximately 30% of the plate, a 0.5% FBS–supplemented medium was used for the 1‐day starvation treatment. The culture fluid was replaced with complete medium and treated with IMQ (10 ng/mL; Sigma‐aldrich, Missouri, USA), rhTNFSF14 (recombinant human TNFSF14, 500 ng/mL; PEPROTECH, New Jersey, USA), rhTNFSF14 (500 ng/mL) + parthenolide (PTL, NF‐κB inhibitor, 2 nM; Selleck, Texas, USA), or rhTNFSF14 (500 ng/mL) + HARMINE (HAR, TWIST1 inhibitor, 50 nM; Selleck, Texas, USA). Total RNA and protein were extracted after 2 and 2.5 days of treatment, respectively.

### 2.4 Bioinformatics Prediction

Human PS dataset GSE13355 was obtained from the Gene Expression Omnibus database, while Rstudio (R4.3.2) was used to analyse the differences in TNFSF14 expression between healthy and psoriatic skin tissues.

### 2.5 Western Blotting

Total protein was extracted from HaCaT cells and mouse skin tissues using protein extraction buffer (PMSF:T‐PER = 1:100). Sodium dodecyl sulfate–polyacrylamide gel electrophoresis was performed at 150 V and the proteins transferred onto polyvinylidene difluoride membranes at 150 mA for 75 min. The membranes were then incubated overnight at 4°C with primary antibodies [rabbit anti‐TNFSF14 (BIOSS, Beijing, China), proliferative cell nuclear antigen (PCNA), MYCN, BAX, BCL2, C‐Caspase3, KRT6, KRT14, KRT17 (Proteintech, Wuhan, China), P‐TAK1, TAK1, P‐p38, p38 (CST, Massachusetts, USA), P‐p65, and p65 (HuaBio, Hangzhou, China) antibodies and mouse anti‐LTβR, HVEM (Santa Cruz, California, USA), and TWIST1 (CST, Massachusetts, USA); all diluted to 1:1000], followed by incubation with horseradish peroxidase‐conjugated goat anti‐rabbit/mouse IgG secondary antibodies (diluted to 1:3000, Servicebio, Wuhan, China) for 1 h at 37°C. The immunoblots were visualised using an enhanced chemiluminescence western blot detection system with β‐actin (rabbit anti‐β‐actin antibody diluted to 1:4000; CST, Massachusetts, USA) as the loading control.

### 2.6 Real‐Time Quantitative Polymerase Chain Reaction

Total RNA was extracted from the HaCaT cells and mouse skin tissue according to the TRIZOL reagent kit (Takara, Kyoto, Japan) instructions and reverse‐transcribed into cDNA. Real‐time quantitative polymerase chain reaction was used to detect the mRNA levels of target genes (see primer sequences in Tables 1 and 2; Sangon Biotech, Shanghai, China). All samples were measured in triplicate. The differences in gene expression were normalised to β‐actin and calculated using the 2^−ΔΔCT^ method.

**TABLE 1:** Primer sequences for real‐time quantitative PCR (mouse).

Gene	Forward primer (5′ → 3′)	Reverse primer (5′ → 3′)
TNFSF14	TGGCTCCTGTAAGATGTGCTG	GTTTCTCCTGAGACTGCATCAA
LTβR	TGCATACCGCAAAGACAAACTC	TGGTGCCCCCTTATCGCATA
HVEM	ACTCGTCTCCCACAAGGAACT	CAGGCCCCTACAGACAACAC
IL‐17A	TTTAACTCCCTTGGCGCAAAA	CTTTCCCTCCGCATTGACAC
IL‐23A	ATGCTGGATTGCAGAGCAGTA	ACGGGGCACATTATTTTTAGTCT
TNF‐α	TCTTCTCATTCCTGCTTGTGG	GGTCTGGGCCATAGAACTGA
IFN‐γ	ATGAACGCTACACACTGCATC	CCATCCTTTTGCCAGTTCCTC
MCP‐1	CAGGTCCCTGTCATGCTTCT	GAGTGGGGCGTTAACTGCAT
IL‐1α	CAAACTGATGAAGCTCGTCA	TCTCCTTGAGCGCTCACGAA
IL‐1β	TCTTTGAAGTTGACGGACCC	TGAGTGATACTGCCTGCCTG
IL‐6	ACAGAAGGAGTGGCTAAGGA	AGGCATAACGCACTAGGTTT
β‐Actin	AGG CCA ACC GTG AAA AGA TG	TGGCGTGAGGGAGAGCATAG

**TABLE 2:** Primer sequences for real‐time quantitative PCR (human).

Gene	Forward primer (5′ → 3′)	Reverse primer (5′ → 3′)
MYCN	TGATCCTCAAACGATGCCTTC	GGACGCCTCGCTCTTTATCT
PCNA	ATGTTCGAGGCGCGCCTGGTC	CTAAGATCCTTCTTCATCCTC
BAX	CAGGATGCGTCCACCAA	AGTAGAAGAGGGCAACCAC
BCL2	CCCTGGCATCTTCTCCTTC	AGAGTTCCTCCACCACCGT
Caspase3	TGGAACAAATGGACCTGTTGACC	AGGACTCAAATTCTGTTGCCACC
KRT6	GGGTTTCAGTGCCAACTCAG	CCAGGCCATACAGACTGCGG
KRT14	TCTGAACGAGATGCGTGACC	TGAAGAACCATTCCTCGGCA
KRT17	GGTGGGTGGTGAGATCAATGT	CGCGGTTCAGTTCCTCTGTC
IL‐17A	TCCCACGAAATCCAGGATGC	GGATGTTCAGGTTGACCATCAC
IL‐23A	CTCAGGGACAACAGTCAGTTC	ACAGGGCTATCAGGGAGCA
TNF‐α	AGCTGGTGGTGCCATCAGAGG	TGGTAGGAGACGGCGATGCG
IFN‐γ	TCGGTAACTGACTTGAATGTCCA	TCGCTTCCCTGTTTTAGCTGC
MCP‐1	CAGCCAGATGCAATCAATGCC	TGGAATCCTGAACCCACTTCT
IL‐1α	TGGTAGTAGCAACCAACGGGA	ACTTTGATTGAGGGCGTCATTC
IL‐1β	CCAGGGACAGGATATGGAGCA	TTCAACACGCAGGACAGGTACAG
IL‐6	AAGCCAGAGCTGTGCAGATGAGTA	TGTCCTGCAGCCACTGGTTC
IL‐8	GTCCTTGTTCCACTGTGCCT	GCTTCCACATGTCCTCACAA
β‐Actin	TCATGAAGTGTGACGTGGACATC	CAGGAGGAGCAATGATCTTGATCT

### 2.7 Tissue Pathological Analysis and Cellular Immunofluorescence

The mouse and human skin tissues were fixed, embedded, sectioned, and baked at 65°C for 3 h. The sections were deparaffinised in xylene, hydrated in alcohol and water, and immersed in citrate buffer at 98°C for 15 min. Tissues (or cells affixed to coverslips) were treated with 3% hydrogen peroxide in the dark for 30 min and blocked with 5% bovine serum albumin for 1 h. The membranes were incubated overnight at 4°C with primary antibodies (diluted to 1:200). Following a wash, the samples were incubated with HRP (Servicebio, Wuhan, China)/alkaline phosphatase (CST, Massachusetts, USA)/CY3 (Bioworld, Minnesota, USA)‐conjugated goat anti‐rabbit/mouse secondary antibodies (diluted to 1:400) for 1 h at 37°C. The molecules of interest were detected using 3,3′‐diaminobenzidine (or an alkaline phosphatase detector), nuclei stained with haematoxylin (or 4′,6‐diamidino‐2‐phenylindole), and cells visualised under a microscope.

### 2.8 Cell Migration Assay

HaCaT cells were seeded into six‐well cell culture plates and evenly scratched with a sterile pipette tip upon reaching approximately 90% confluence. Each well was washed with phosphate buffered saline and then supplemented with MEM containing 0.5% FBS, followed by treatment with this matrix under different conditions. The scratched images were observed under a microscope and captured after 0 and 48 h of treatment.

### 2.9 Measuring Skin Inflammation

PS Area and Severity Index (PASI) scores were used to assess psoriatic lesion severity, including scaling, thickness, and erythema, on a scale from 0 to 4 (0‐none, 1‐slight, 2‐moderate, 3‐marked, 4‐very marked).

### 2.10 Statistical Analysis

Statistical analyses were performed using GraphPad software (version 8.0). Groups were analysed using a *t*‐test or one‐way analysis of variance with Tukey's multiple comparison test. Statistical significance was set at *p* < 0.05.

## 3 Results

### 3.1 Upregulation of TNFSF14 and HVEM/LTβR Expression in Epidermis of Psoriatic Lesions

**FIGURE 1:**
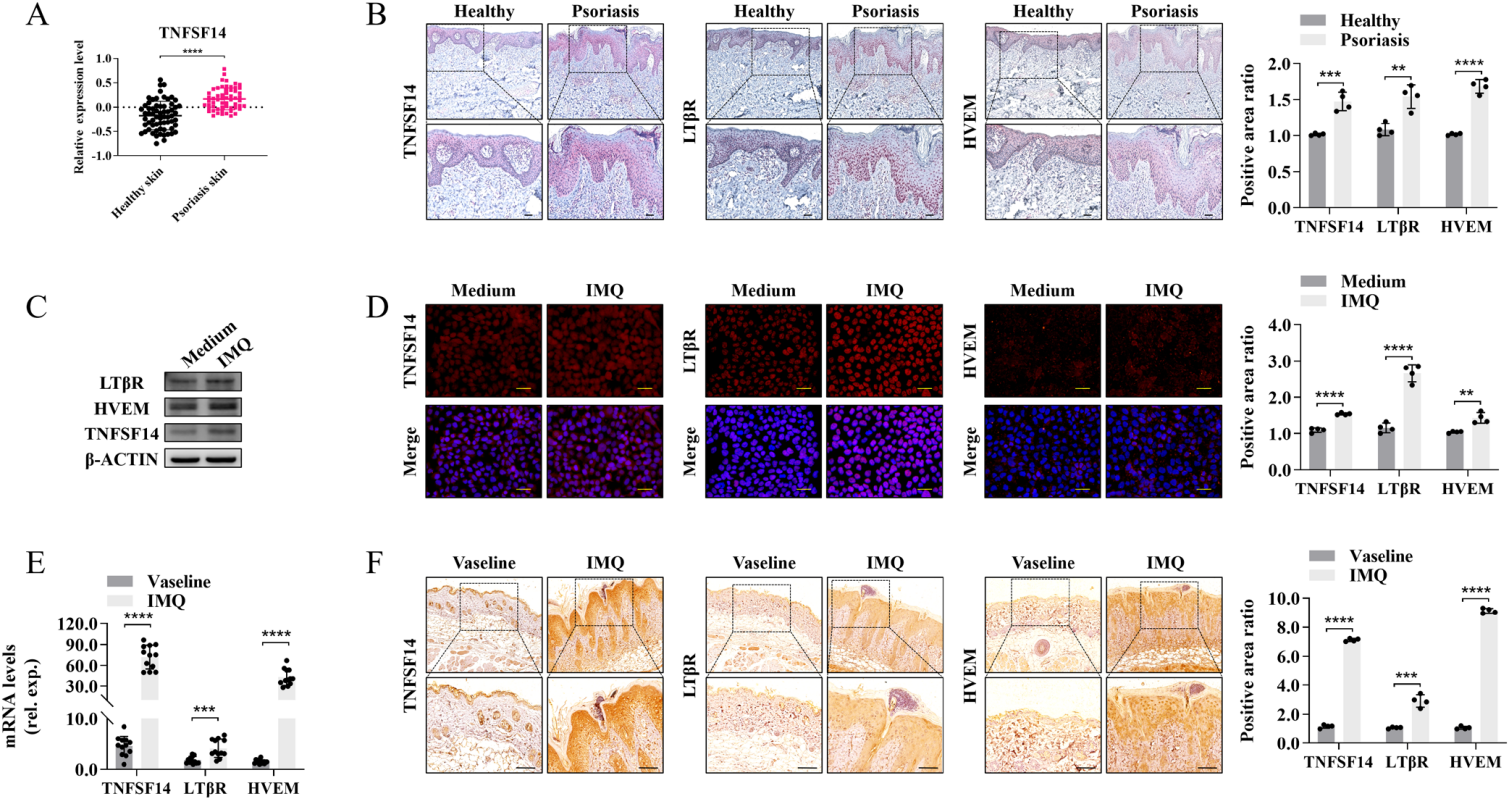
Increased expression of TNSF14 and its two receptors LTβR and HVEM during PS development. (A) Expression of TNFSF14 in human psoriatic and normal skin tissue in the GEO dataset GSE13355; (B) IHC staining for TNFSF14 and LTβR/HVEM in human psoriatic skin lesions and healthy controls. Scale bar = 100 μm; (C and D) Human immortalised KCs (HaCaT) were treated with IMQ (10 ng/mL) for 60 h, and the expression of TNSF14 and LTβR/HVEM were tested by Western blot (C) and IF staining (D). Scale bar = 25 μm; (E and F) TNFSF14^+/+^ mice were treated with IMQ (62.5 mg/day) or Vasline for 5 days, skin samples were analysed with qRT‐PCR (E) and IHC (F) for the expression of TNFSF14 and LTβR/HVEM. Right panel: Semi‐quantification for positive staining area. Scale bar = 100 μm (*p < 0.05; **p < 0.01; ***p < 0.001; ****p < 0.0001).

To explore the relationship between TNFSF14 and PS, we analysed published RNA sequencing data and found higher TNFSF14 mRNA levels in psoriatic lesions from patients versus healthy volunteers (Figure 1A). Further immunohistochemistry (IHC) analyses revealed a significant escalation of both TNFSF14 and HVEM/LTβR expression in the epidermal layer of psoriatic skin (Figure 1B), confirming the possible correlation between the TNFSF14 pathway and KCs during PS development. Similar results were observed in the IMQ‐treated HaCaT cells (Figure 1C,D). Moreover, consistent trends in the expression and localisation of TNFSF14 and its receptors were observed in IMQ‐induced psoriatic skin lesions in mice (Figure 1E,F). These observations indicate that the TNFSF14‐HVEM/LTβR pathway is possibly closely associated with the development of PS.

### 3.2 TNFSF14 Deficiency Ameliorates IMQ‐Induced Psoriatic Skin Inflammation in Mice

**FIGURE 2:**
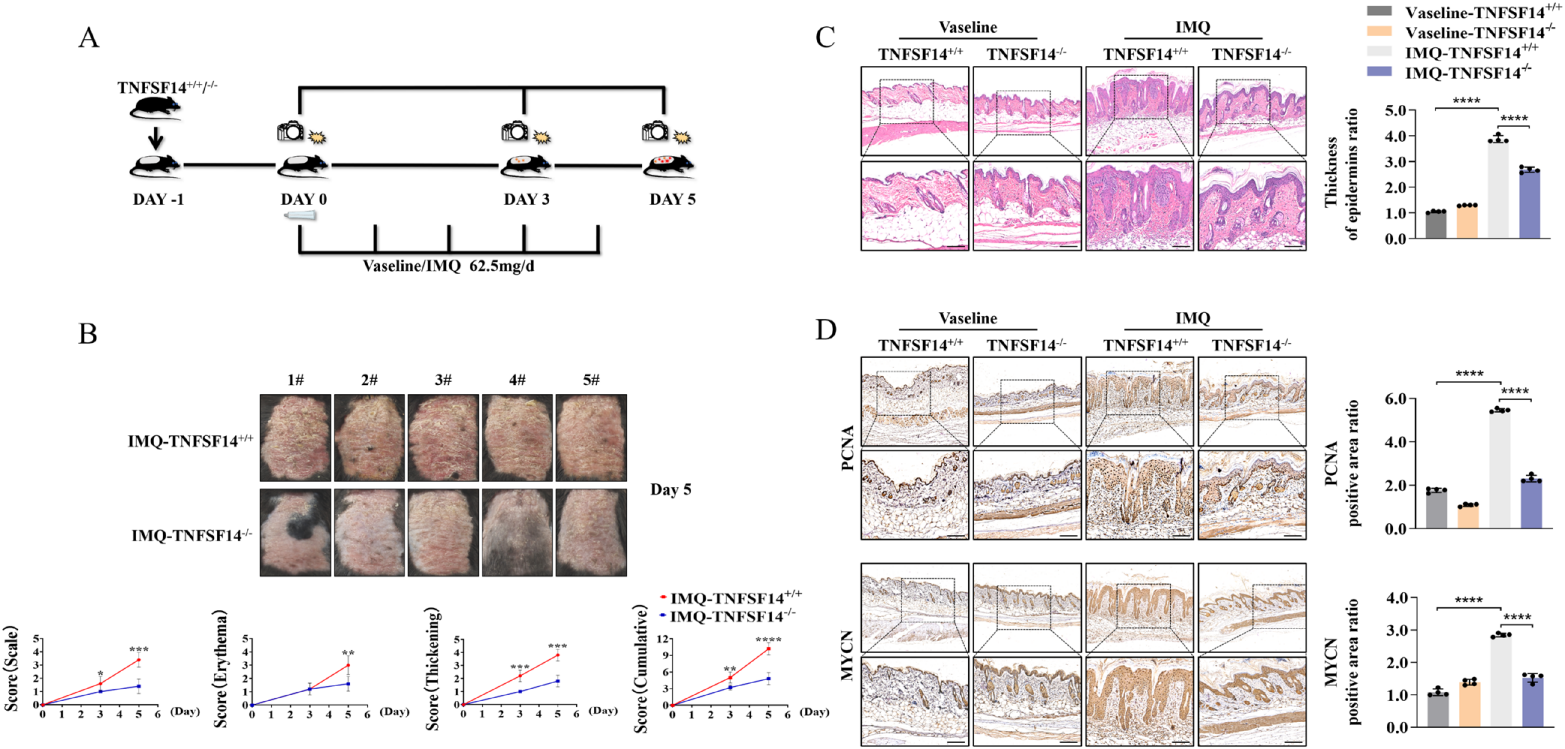
TNFSF14 deficiency attenuates IMQ‐induced psoriatic skin inflammation. (A) Protocols of IMQ‐induced psoriatic skin inflammation; (B) Macroscopic phenotype of psoriatic lesions in TNFSF14^+/+^ and TNFSF14^−/−^ mice treated with IMQ (62.5 mg/day). Below: Back skin disease severity was measured daily based on the mPASI. Data are presented as the mean ± SD (*n* = 5 for each group); (C) H&E staining for back skin sections. Right panel: Semi‐quantification for epidermal thickness. Scale bar = 100 μm; (D) IHC staining and semi‐quantification for PCNA and MYCN. Scale bar = 100 μm (*p < 0.05; ***p < 0.001; ****p < 0.0001).

To further explore the role of TNFSF14 signalling in PS development, we used TNFSF14 gene knockout mice. Compared to TNFSF14^+/+^ mice, TNFSF14^−/−^ mice showed a remarkable reduction in IMQ‐induced psoriatic skin inflammation with ameliorated crust formation, attenuated scale severity, and decreased cumulative modified PASI (mPASI) scores (Figure 2A,B). Consistently, histological analyses revealed increased thickening of the epidermal layer in IMQ‐ versus vehicle‐treated TNFSF14^+/+^mice; however, this effect was notably mitigated upon TNFSF14 deficiency (Figure 2C). Dysregulated KC hyperproliferation contributes to epidermal thickness in PS [28], so we further detected the expressions of PCNA [29] and MYCN [30, 31] in skin lesions using IHC staining. In accordance with the histological findings, PCNA and MYCN expressions were significantly up‐regulated after IMQ stimulation, whereas TNFSF14 loss clearly decreased their expression levels, indicating remarkably decreased KC hyperproliferation in TNFSF14^−/−^ mice (Figure 2D). Taken together, TNFSF14 deficiency significantly attenuated the development of PS and KC proliferation in IMQ‐induced psoriatic dermatitis.

### 3.3 TNFSF14 Loss Inhibits Keratinisation and KC Apoptosis During Development of IMQ‐Induced Psoriatic Dermatitis in Mice

**FIGURE 3:**
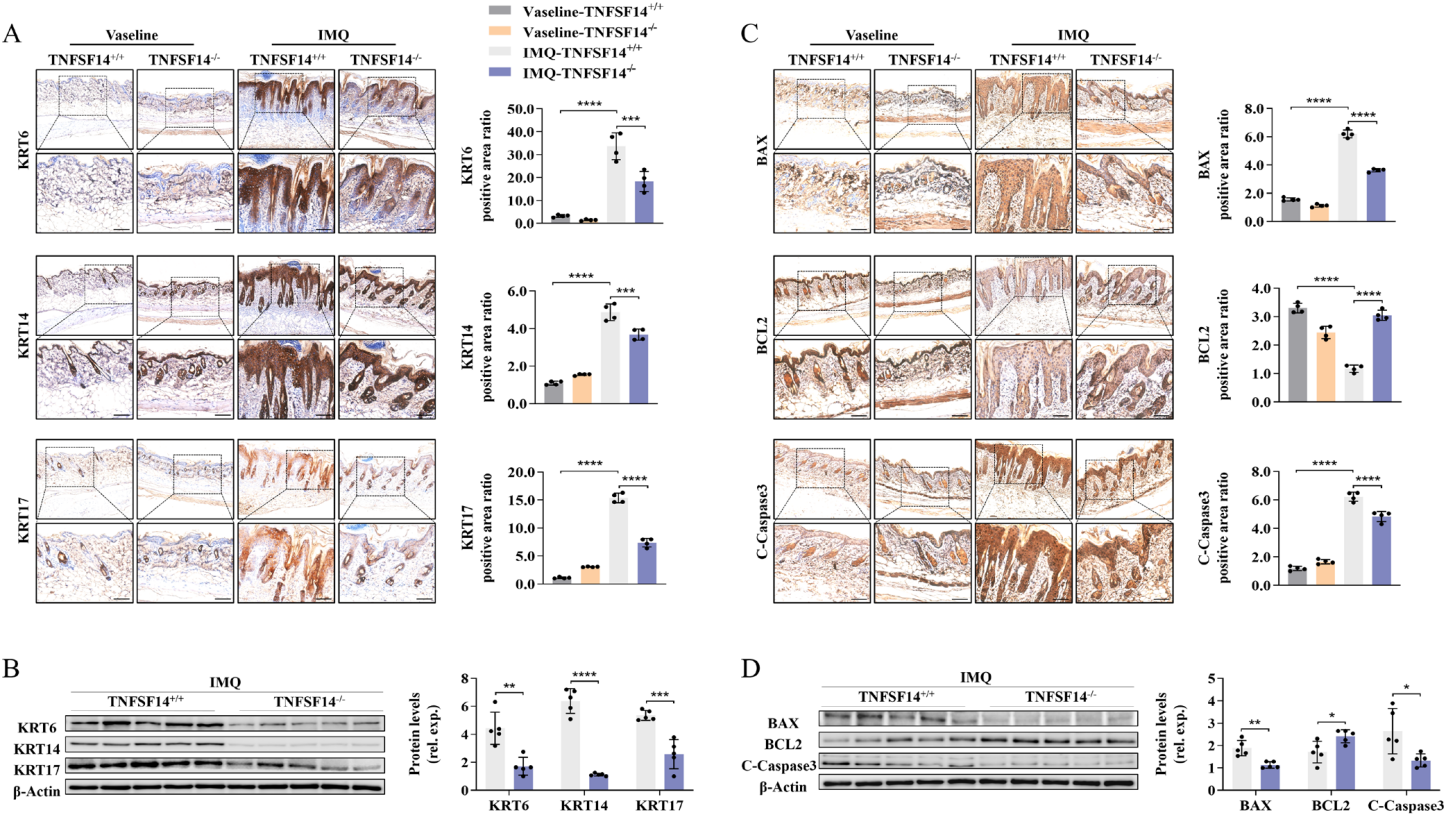
TNFSF14 deficiency ameliorates IMQ‐induced hyper‐keratinization and apoptosis in psoriatic skin lesion of mice. (A) IHC staining for KRT6, KRT14 and KRT17 expression. Scale bar = 100 μm; (B) Western blot analysis for the KRT6, KRT14 and KRT17 expression; (C) IHC staining for the expression of apoptosis‐related molecules (BAX, BCL2, C‐Caspase3). Scale bar = 100 μm; (D) Western blot analysis for the apoptosis‐related molecules (BAX, BCL2, C‐Caspase3) expression. Right: Semi‐quantification for protein levels (*p < 0.05; **p < 0.01; ***p < 0.001; ****p < 0.0001).

Keratin (KRT), the major skeletal component of KCs, drives keratinisation, proliferation, and inflammatory response while controlling skin firmness or looseness [32]. To explore the effect of TNFSF14 on keratinisation, next we analysed the expression of three PS‐related KRT (KRT6/14/17) [14, 32]. The application of IMQ versus vehicle remarkably increased the expressions of these KRT, while TNFSF14 deficiency clearly reversed this effect (Figure 3A,B). Previous evidence suggests that skin cell apoptosis mediates the pathogenesis of IMQ‐induced psoriasiform dermatitis [15]. To determine the effect of TNFSF14 on cell apoptosis, we tested three essential apoptosis‐related molecules (BAX, BCL2, and Cleaved‐Caspase3) [33]. IMQ treatment dramatically increased proapoptotic BAX and C‐Caspase3 expression but down‐regulated BCL2 anti‐apoptotic protein expression, and TNFSF14 loss significantly counteracted these effects (Figure 3C,D). Overall, the TNFSF14 knockout attenuated keratinisation and apoptosis in mice with IMQ‐induced psoriasiform dermatitis.

### 3.4 Deletion of TNFSF14 Protects IMQ‐Induced Psoriatic Skin Inflammation in Mice

**FIGURE 4:**
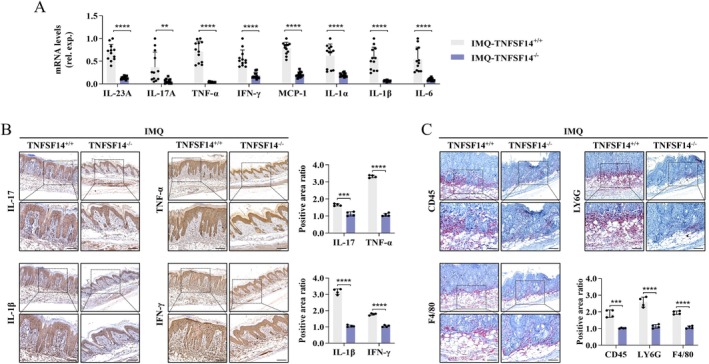
TNFSF14 is required for IMQ‐induced inflammatory cytokines expression and immune cells infiltration in psoriatic skin lesion of mice. Following 5 days of IMQ treatment, back skin tissues from TNFSF14^+/+^ and TNFSF14^−/−^ mice were collected. (A) Quantitative RT‐PCR analysis for the indicated inflammatory cytokines genes. Data are presented as the mean ± SD. (B) IHC staining for the indicated inflammatory cytokines. Right: Semi‐quantification for protein levels. Scale bar = 100 μm; (C) IHC staining for the immune cell infiltration in the psoriatic skin lesion. Scale bar = 100 μm (**p < 0.01; ***p < 0.001; ****p < 0.0001).

The IL‐23/IL‐17 axis is an essential mediator in PS pathology [34]. As shown in Figure 4A, compared to TNFSF14^+/+^ mice, TNFSF14 deficiency significantly down‐regulated the IL‐23A/IL‐17Aaxis‐related cytokine mRNA levels in psoriatic skin lesions. Similar results were consistently found in IL‐17, tumour necrosis factor‐α (TNF‐α), interferon‐gamma (IFN‐γ), and IL‐1β protein levels by IHC staining (Figure 4B). Moreover, skin infiltrating mononuclear cells (CD45^+^ cells), neutrophils (Ly‐6G^+^ cells), and macrophages (F4/80^+^ cells) were dramatically reduced in TNFSF14^−/−^ mice (Figure 4C). Taken together, TNFSF14 deletion ameliorated IMQ‐induced IL‐23/IL‐17 cytokine production and inflammatory cell infiltration.

### 3.5 Exogenous Recombinant TNFSF14 Promotes KC Abnormalities

**FIGURE 5:**
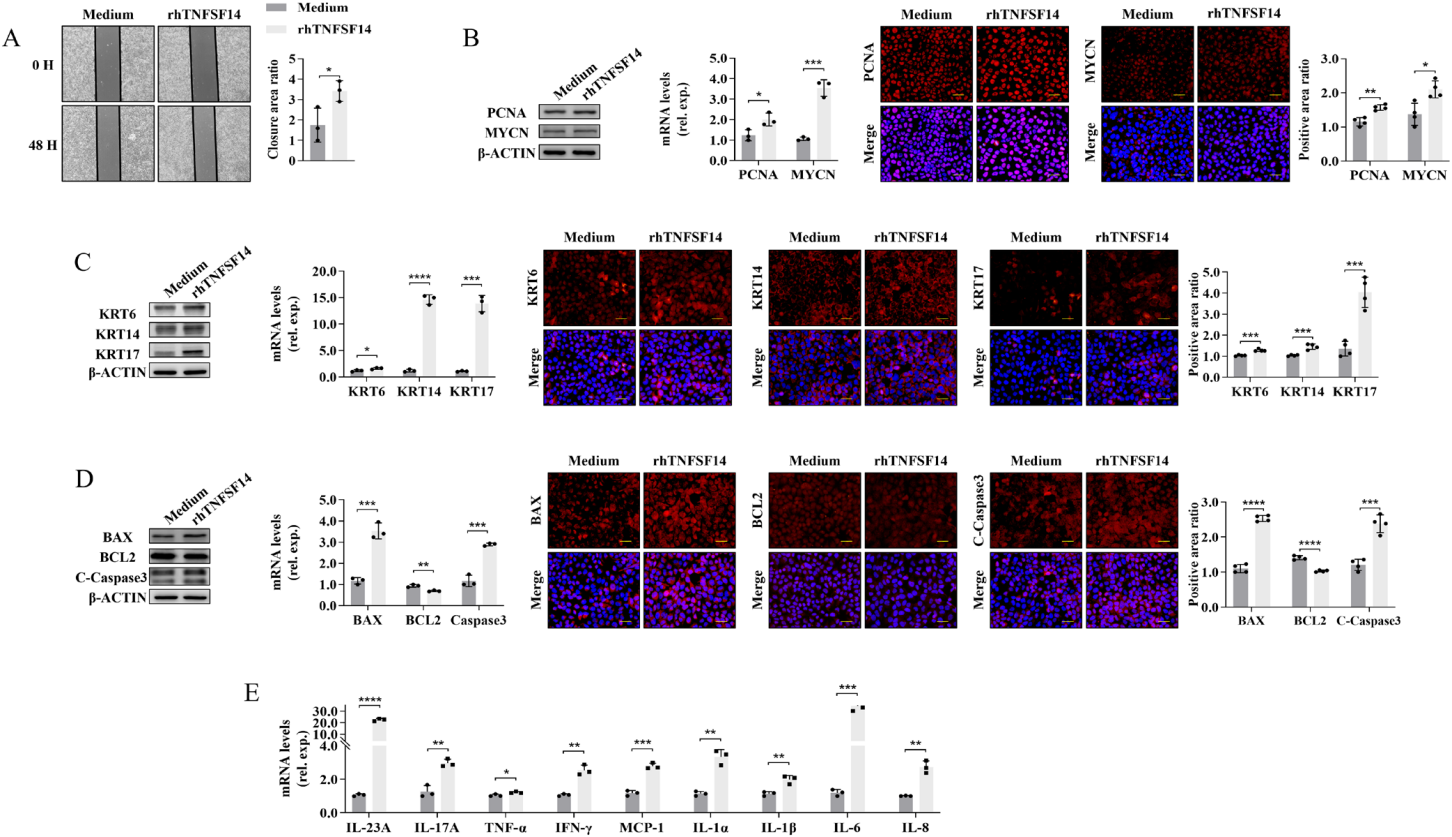
rhTNFSF14 enhances KCs disorders in vitro. HaCaT cells were stimulated with recombined human TNFSF14 (rhTNFSF14, 500 ng/mL) or medium for 60 h. (A) Cells proliferation was measured with cell wound scratch assay; (B–D) PCNA and MYCN expression (B), cells hyper‐keratinization (C) and cells apoptosis (D) were assayed by western blot, quantitative RT‐PCR and IF staining. Scale bar = 25 μm; (E) Indicated cytokines expression were measured by quantitative RT‐PCR. Data are presented as the mean ± SD (*p < 0.05; **p < 0.01; ***p < 0.001; ****p < 0.0001).

Next, we examined the in vitro effects of exogenous TNFSF14 on KC abnormalities in HaCaT cells. As shown in Figure 5A, stimulation with recombinant human TNFSF14 (rhTNFSF14) notably enhanced the relative area ratio of cell scratch wound closure. Meanwhile, the protein and mRNA levels of the proliferation‐related molecules PCNA and MYCN (Figure 5B) and KRT (KRT6, KRT14, and KRT17) (Figure 5C) were significantly up‐regulated upon rhTNFSF14 stimulation. Moreover, rhTNFSF14 significantly enhanced the expressions of the pro‐apoptotic molecules BAX and C‐Caspase3 while decreasing the expression of the anti‐apoptotic molecule BCL2 (Figure 5D). Furthermore, rhTNFSF14 stimulation significantly up‐regulated the mRNA levels of the IL‐23A/IL‐17A axis–related inflammatory cytokines (Figure 5E). These findings suggest that TNFSF14 can induce certain in vitro characteristics similar to those of PS. Specifically, it triggers KC abnormalities including cellular proliferation, keratinisation, apoptosis, and inflammatory responses.

### 3.6 TNFSF14 Stimulation Enhances KC Abnormalities via NF‐κB Signalling

**FIGURE 6:**
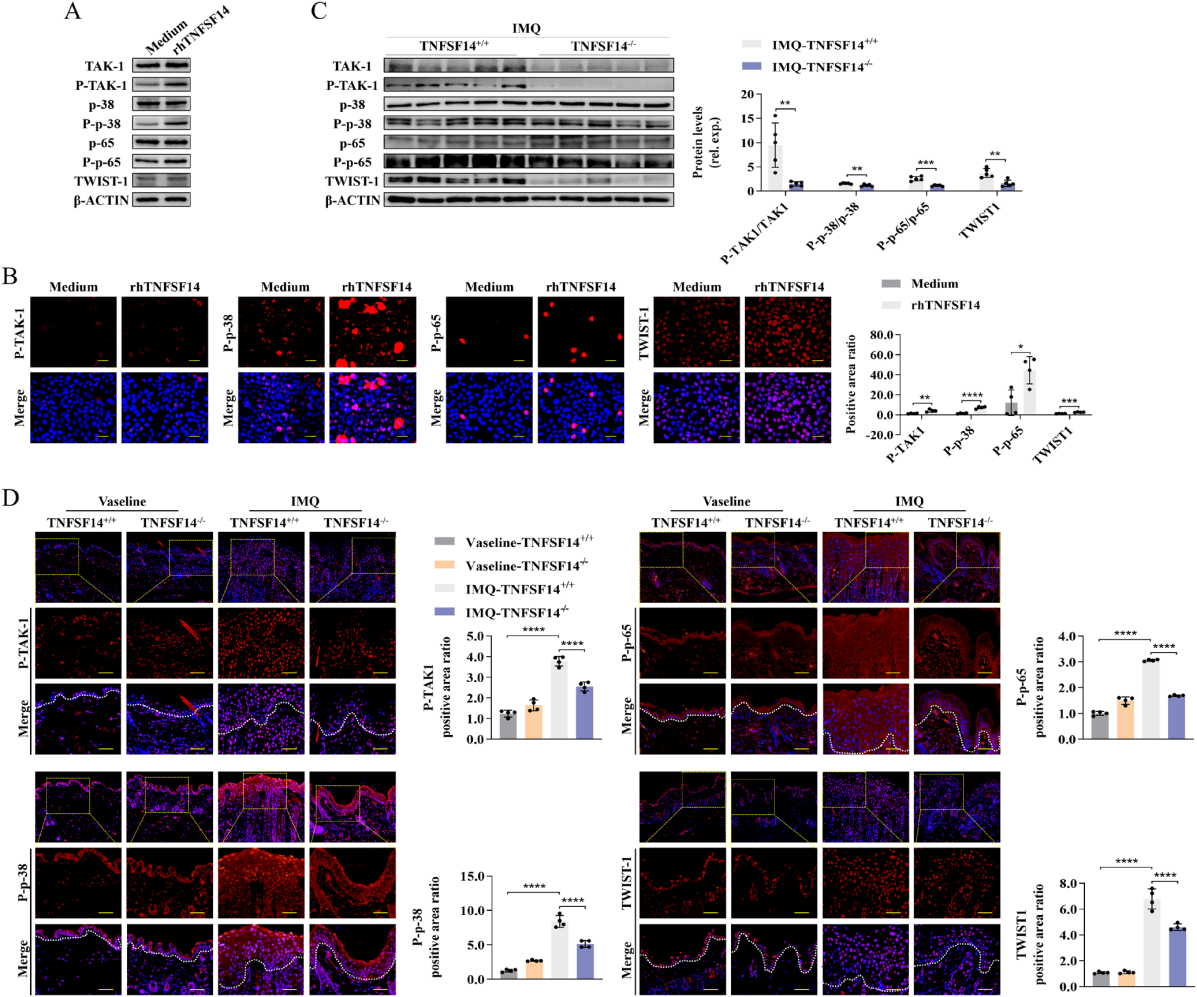
TNFSF14 participates in the activation of TAK‐1/p38/NF‐κB/TWIST1 pathway. (A and B) HaCaT cells were treated with rhTNFSF14 (500 ng/mL) for 60 h. TAK‐1/p38/NF‐κB/TWIST1 activation was assayed with western blot (A) and IF staining (B). Scale bar = 25 μm; (C and D) After IMQ treatment or Vasline stimulation for 5 days, back skin samples of mice were collected. TAK‐1/p38/NF‐κB/TWIST1 pathway activation was measured with western blot (C) and IF staining (D). Right: Semi‐quantification for protein levels. Scale bar = 50 μm (*p < 0.05; **p < 0.01; ***p < 0.001; ****p < 0.0001).

**FIGURE 7:**
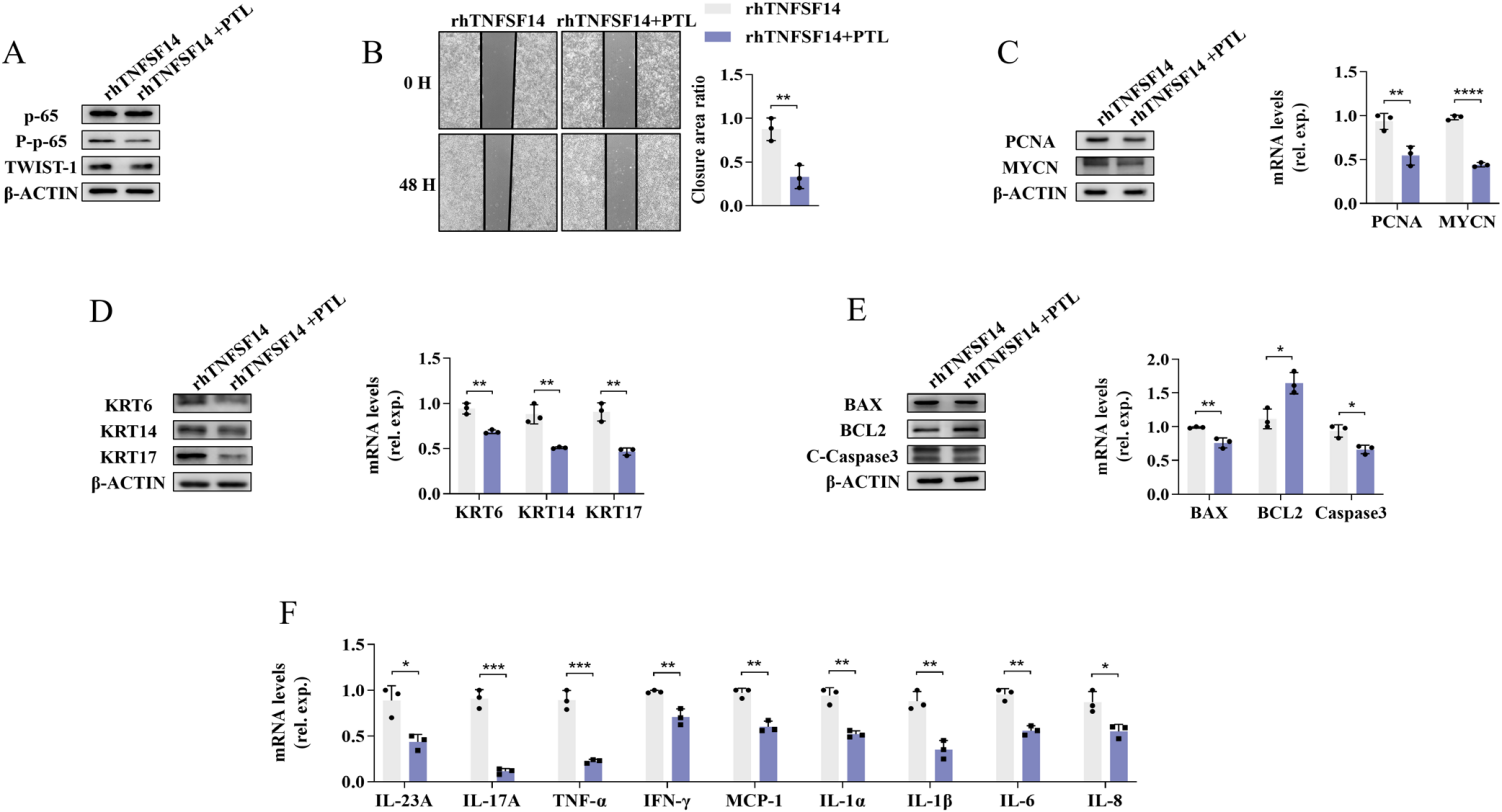
TNFSF14 mediates KCs abnormalities via enhancing the NF‐κB signalling activation. HaCaT cells were treated with rhTNFSF14 (500 ng/mL) alone or rhTNFSF14 plus NF‐κB activation inhibitor Parthenolide (PTL, 2 nM) for 60 h and cells were collected. (A) P‐p65 and TWIST1 expression were measured by western blot; (B) Cells proliferation with cell wound scratch assay; (C) PCNA and MYCN expression, (D) Cells hyper‐keratinization and (E) Cells apoptosis was assayed with western blot and quantitative RT‐PCR; (F) Quantitative RT‐PCR results for indicated inflammatory cytokines expression. Data are presented as the mean ± SD (*p < 0.05; **p < 0.01; ***p < 0.001; ****p < 0.0001).

NF‐κB pathway activation promotes the production of chemokines and pro‐inflammatory factors and participates in various physiological processes of cells in inflammatory‐associated diseases [35, 36]. As shown in Figure 6A,B, the relative phosphorylated expression levels of p65 (P‐p65)/p65 and its upstream molecules TAK1 (P‐TAK1)/TAK1 and p38 (P‐p38)/p38 were all significantly up‐regulated upon stimulation with rhTNFSF14 in HaCaT cells. The phosphorylated levels of these molecules in IMQ‐treated TNFSF14^−/−^ mice were remarkably down‐regulated compared to those in IMQ‐treated TNFSF14^+/+^ mice (Figure 6C,D). These results suggest that TNFSF14 signalling may positively modulate NF‐κB pathway activation during psoriatic dermatitis development. To further validate the effect of NF‐κB activation in TNFSF14‐primed KC abnormalities, we blocked the NF‐κB pathway by its specific inhibitor PTL, which notably diminished rhTNFSF14‐enhanced NF‐κB activation as evidenced by decreased P‐p65 expression (Figure 7A). Additionally, PTL clearly ameliorated rhTNFSF14‐primed KC proliferation, improved cell scratch wound closure (Figure 7B), and increased PCNA and MYCN expression (Figure 7C). Consistent with this, the enhanced expression of KRT, apoptosis, and inflammatory cytokines in KCs induced by rhTNFSF14 was remarkably attenuated upon PTL application (Figure 7D–F). Collectively, these findings suggest that TNFSF14 promotes KC abnormalities via the TAK1/p38/NF‐κB signalling pathway.

### 3.7 TWIST‐1 Involved in TNFSF14‐Primed NF‐κB Signalling Activation–Mediated KC Abnormalities

**FIGURE 8:**
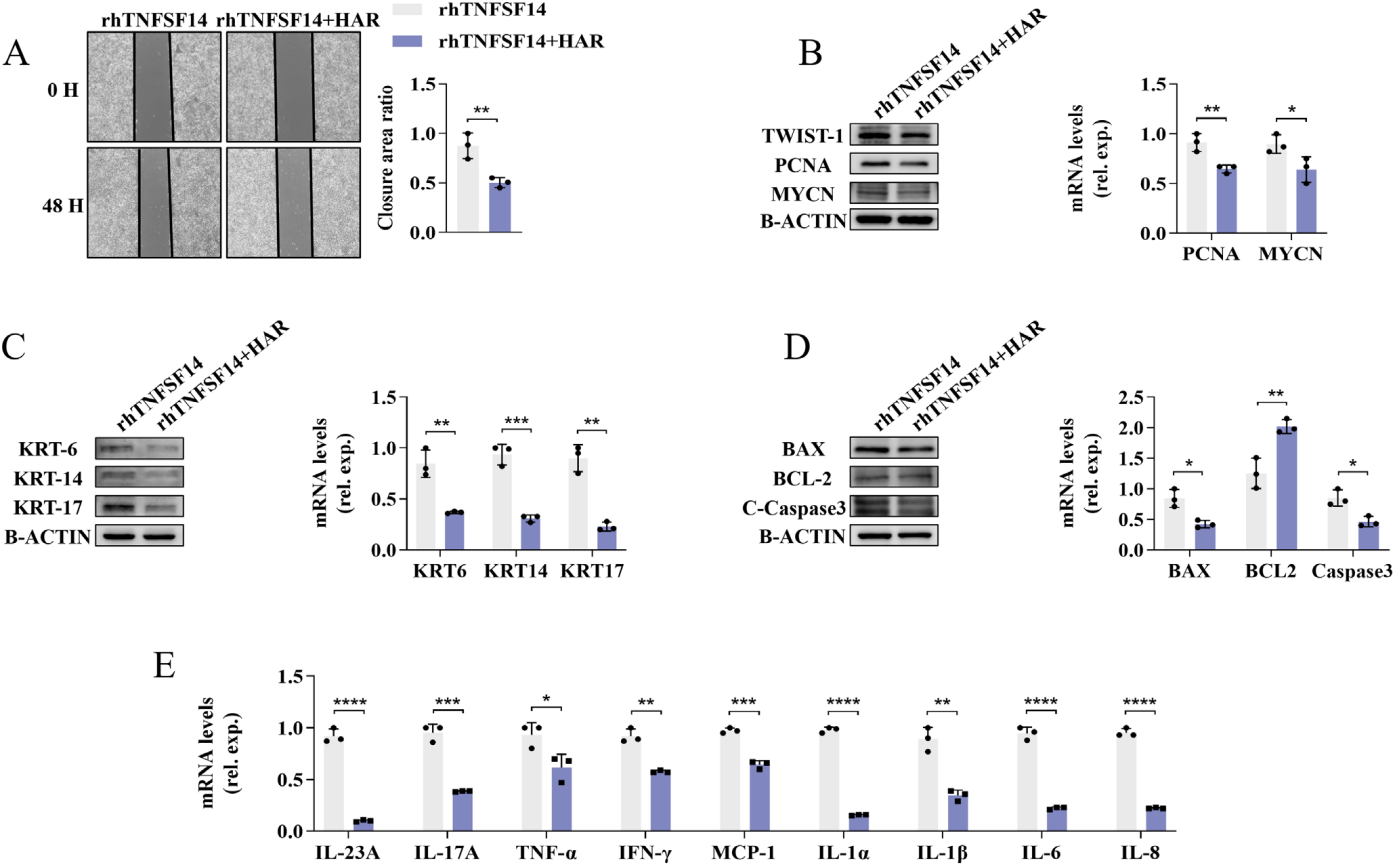
TNFSF14 promotes KCs dysfunction with TWIST1 signalling dependent. HaCaT cells were treated with rhTNFSF14 (500 ng/mL) alone or rhTNFSF14 plus TWIST1 inhibitor HARMINE (HAR, 50 nM) for 60 h and cells were collected. (A) Cells proliferation was measured with cell wound scratch assay; (B) PCNA and MYCN expression, (C) Cells hyper‐keratinization and (D) Cells apoptosis was assayed with western blot and quantitative RT‐PCR; (E) Quantitative RT‐PCR analysis for indicated inflammatory cytokines. Data are presented as the mean ± SD (*p < 0.05; **p < 0.01; ***p < 0.001; ****p < 0.0001).

TWIST1 is a highly conserved transcription factor containing a basic helix–loop–helix domain. Upon becoming activated by NF‐κB signalling (Figure 7A), it participates in multiple essential biological processes, such as programmed cell death [37], epithelial–mesenchymal transition [38], and inflammatory responses [39]. TWIST1‐specific inhibitor HARMINE (HAR) was used to uncover the role of TWIST1 in TNFSF14‐induced KC abnormalities. As shown in Figure 8A,B, this inhibition remarkably decreased TNFSF14‐induced KC proliferation, reduced cell scratch wound closure, and down‐regulated PCNA and MYCN expressions. Correspondingly, the increased expression of KRT induced by TNFSF14 was down‐regulated in the HAR‐treated group (Figure 8C). Similarly, inhibiting TWIST1 activation with HAR markedly ameliorated TNFSF14‐induced KC apoptosis and inflammatory cytokine expression (Figure 8D,E). Overall, these results suggest that the TAK1/p38/NF‐κB/TWIST1 signalling pathway is involved in TNFSF14‐mediated KC abnormalities.

### 3.8 Blocking TNFSF14 Signalling With Soluble Receptor Fusion Proteins Improves IMQ‐Induced KC Keratinisation and Psoriasiform Skin Inflammation in Mice

**FIGURE 9:**
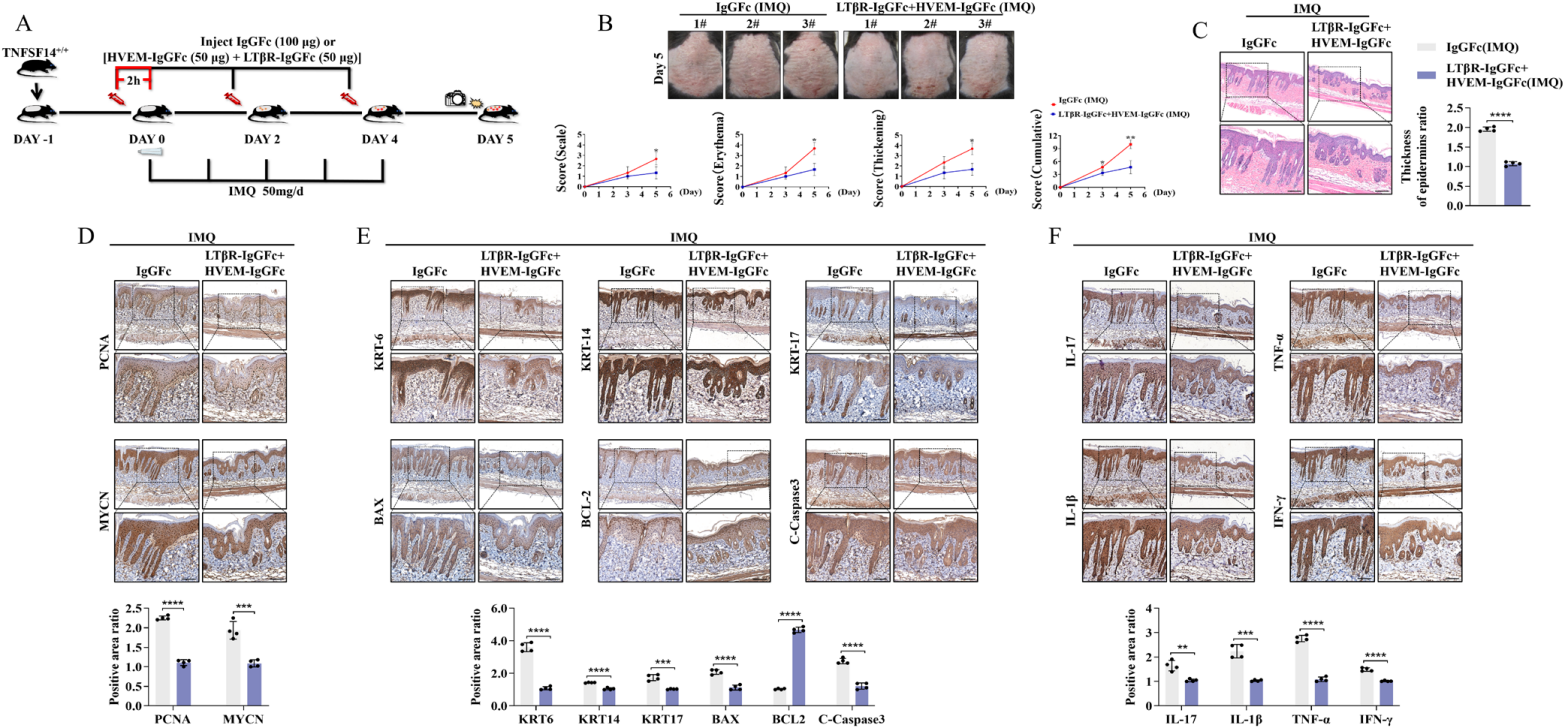
Blockage of TNFSF14 with its soluble receptors fusion proteins HVEM‐Ig and LTβR‐Ig attenuates IMQ‐induced psoriatic skin inflammation. (A) Protocols of fusion protein application and IMQ‐induced psoriatic skin inflammation; (B) Macroscopic phenotype of psoriatic lesions in TNFSF14^+/+^ mice after treatment with fusion proteins (50 mg/day). Below: Back skin disease severity was measured daily based on the mPASI. Data are presented as the mean ± SD (*n* = 3 for each group); (C) H&E staining for back skin sections. Right panel: Semi‐quantification for epidermal thickness. Scale bar = 100 μm; (D) PCNA and MYCN expression, (E) Hyper‐keratinization and apoptosis, and (F) The expression of indicated inflammatory cytokines were assayed with IHC staining and semi‐quantification. Scale bar = 100 μm (*p < 0.05; **p < 0.01; ***p < 0.001).

Finally, to validate the function of TNFSF14 signalling in the pathogenesis of PS in vivo, we blocked this pathway using its soluble receptors fusion proteins LTβR‐IgGFc and HVEM‐IgGFc (Figure 9A). We found that blocking TNFSF14 signalling significantly reduced IMQ‐induced psoriatic skin dermatitis similar to that seen in TNFSF14^−/−^ mice (Figure 9B). Correspondingly, blocking TNFSF14 signalling remarkably alleviated hyperkeratosis and thickening of the epidermal layer (Figure 9C). Moreover, IMQ‐primed hyperproliferation, hyperkeratosis, apoptosis, and excessive inflammatory cytokine expression were significantly ameliorated in LTβR‐IgGFc‐ and HVEM‐IgGFc‐treated mice (Figure 9D–F). In conclusion, TNFSF14 mediates KC abnormalities and IMQ‐induced psoriatic skin inflammation by interacting with its receptors. Thus, targeting TNFSF14 may be a promising strategy for the treatment of patients with PS.

## 4 Discussion

Accumulating studies have revealed that KCs are both executors of immune responding and strong triggers in the pathological progression of PS [8]. TNFSF14, an essential member of the TNF superfamily, was recently identified as a vital component of several immune diseases [40, 41]. Additionally, TNFSF14‐HVEM signalling promotes inflammatory cytokine production and KC hyperplasia in atopic dermatitis [24]. However, the effects and underlying mechanisms of TNFSF14 in mediating KC abnormalities and IMQ‐induced psoriatic skin inflammation remain not fully understood. Here we found that TNFSF14 and its two receptors were markedly up‐regulated in IMQ‐primed KCs and psoriatic skin sections. Moreover, we demonstrated that TNFSF14 knockout attenuated IMQ‐induced psoriatic skin inflammation accompanied by a significant alleviation in epidermal hyperplasia and significant reduction in KC proliferation, keratinisation, and apoptosis. In contrast, TNFSF14 stimulation significantly enhances KC abnormalities, including increased proliferation and keratinisation, increased apoptosis, and up‐regulated cytokine expression.

A mechanistic investigation further revealed that TNFSF14 signalling–mediated abnormalities occur in KCs via the TAK1/P38/NF‐κB/TWIST1 pathway. Finally, we showed that blocking TNFSF14 signalling with soluble receptor fusion proteins in wild‐type mice phenocopied TNFSF14 knockout mice, which exhibited dramatically ameliorated skin inflammation. Collectively, our study findings indicate that TNFSF14‐HVEM/LTβR mediates KC dysfunction and IMQ‐induced psoriatic skin inflammation by enhancing NF‐κB/TWIST1 signalling.

Increasing evidence regarding several inflammatory diseases suggests that TNFSF14 signalling dysregulation is a critical disease‐driving mechanism, especially in skin disorders [24, 25]. TNFSF14 mediates fibrosis progression in several tissue types, including the skin, lungs, and kidneys, emerging as a promising therapeutic target to halt fibrosis [25, 42, 43]. It was also shown that TNFSF14‐HVEM signalling in KCs promotes the development of atopic dermatitis–like skin inflammation induced by house dust mite allergens [24]. Consistent with these recently published data, our study further confirmed that TNFSF14 is required for IMQ‐induced skin inflammation evidenced by significantly reduced epidermal thickening, scaling, and erythema in TNFSF14 knockout mice. Most significantly, blocking TNFSF14 signalling with soluble receptor fusion proteins clearly reduced IMQ‐induced skin inflammation, further extending its therapeutic applications. Collectively, TNFSF14 is an essential mediator of the progression of PS and may be a promising therapeutic target for its clinical treatment.

KCs, among the major components of epidermal skin, were recently demonstrated as key mediators of PS pathology progression. Specifically, their abnormal biological behaviours (e.g., proliferation, keratinization, apoptosis, and cytokine production) are paramount to the development of PS [9]. The effects of TNFSF14 on KCs are being increasingly explored in inflammatory skin diseases, especially atopic dermatitis and PS, thereby revealing its pathological role in mediating KC proliferation, fibrosis accumulation, periosteal production, and inflammation [24, 25]. Consistently, the present study demonstrated that TNFSF14 knockout or blocking with its soluble receptor fusion proteins significantly decreased PCNA and MYCN expressions, ameliorated apoptosis, and down‐regulated IL‐23/IL‐17 axis cytokine expressions in IMQ‐induced murine PS‐like skin dermatitis. Accordingly, exogenous stimulation with TNFSF14 triggered KC abnormalities as evidenced by enhanced proliferation, KRT expression, increased apoptosis, and up‐regulated inflammatory cytokine production. Taken together, these data suggest that TNFSF14 is critical for KC dysfunction in inflammatory skin disorders, including PS.

TNFSF14 not only promotes KC proliferation, but it also triggers apoptosis via jointly activating IFN‐γ to induce NF‐κB signalling and decrease BCL2 expression [44]. One likely possible explanation for this is that cell death–induced regeneration, a hypothesis that is supported by the observation that a small amount of viable dying tumour cells in radiotherapy or chemotherapy promotes rapid regeneration through Caspase‐3 [45]. As shown in Figure 2, TNFSF14 knockout remarkably reduced skin thickness (proliferation), scale production, and shedding (apoptosis) to maintain relative balance of the skin in a state of “weak activation.”

Our previous studies revealed that TNFSF14 activated the NF‐κB pathway in several immune‐related diseases. For instance, TNFSF14 exacerbated sepsis‐related acute kidney injury via the TLR4/MyD88/NF‐κB pathway [46] and activated the NFκB‐TLR3 pathway to induce acute hepatitis [47]. In accordance with these observations, here we further found that TNFSF14 deficiency markedly suppressed the activation of NF‐κB and its upstream molecules P‐TAK1 and P‐p38 in skin lesions. In contrast, a notable increase in the phosphorylation of TAK‐1, p38, and p65 was observed in HaCaT cells upon stimulation with exogenous recombinant TNFSF14. Additionally, blocking NF‐κB signalling with its specific inhibitor, PTL, partially reversed the KC abnormalities induced by recombinant TNFSF14. Collectively, TNFSF14/TAK1/p38/NFκB signalling enhances KC dysfunction in PS‐related skin inflammation.

Hyperplastic KCs in PS have some abilities, especially aggressive proliferation, similar to cancer cells [48] accompanied by the high expression of proliferation markers including PCNA or MYCN. TWIST1, a pivotal transcriptional enhancer [49], is reportedly critical for the formation and invasiveness of both chemically and ultraviolet B–induced skin tumours in mice. KC‐specific TWIST1 deletion clearly attenuated chemical and ultraviolet B–induced epidermal hyperproliferation [50, 51]. Here we found that TNFSF14 deficiency significantly ameliorated TWIST1 activation in IMQ‐induced psoriatic dermatitis, while additional experiments indicated that exogenous stimuli with TNFSF14 led to opposite effects. Additionally, inhibiting TWIST1 with a specific inhibitor, HAR, clearly decreased KC abnormalities, including proliferation, KRT production, and inflammatory cytokine expression. These data suggest that TNFSF14/NF‐κB/TWIST1 is essential to KC abnormalities and IMQ‐induced skin dermatitis.

A recent single‐cell RNA sequence analysis of skin tissues taken from both patients with PS and healthy individuals revealed that its receptors LTβR/HVEM were strongly expressed in KCs as well as dendritic, Langerhans, and endothelial cells, whereas TNFSF14 was predominantly expressed in T cells, with Langerhans cells and fibroblasts also testing positive and enhanced transcripts of TNFSF14 being identified, but no difference was observed in the expression levels of its receptors in psoriatic skin lesions [26]. In contrast, we found that both TNFSF14 and its two receptors were significantly up‐regulated in IMQ‐primed KCs; importantly, differences in models, cell types, and species may account for this discrepancy. Except for the critical role of TNFSF14‐LTβR/HVEM in KCs, whether other skin tissue resident cells (including T, Langerhans, and dendritic) participate in the pathogenesis of PS remains unclear. Specifically, the conditional deletion of these molecules in T, dendritic, and Langerhans cells could uncover this point in further studies. Our unpublished data show that TNFSF14 deficiency significantly ameliorated IMQ‐induced IL‐17A‐positive γδT cellular expansion in the skin and draining lymph nodes; similar findings were made in Langerhans cells. Moreover, blocking the interaction between TNFSF14‐LTβR and the fusion proteins further ameliorated epidermal hyperplasia and dermal thickness compared to KC‐specific LTβR deletion in IMQ‐induced psoriatic skin inflammation (unpublished data). Overall, these results suggest that TNFSF14 signalling in other resident skin cells may also play an important role in the progression of PS.

## Author Contributions

**Sheng‐jie Long:** conceptualization (equal), data curation (equal), formal analysis (equal), investigation (equal), methodology (equal), software (equal), validation (equal), visualization (equal), writing – original draft (equal), writing – review and editing (equal). **Quan‐you Zheng:** conceptualization (equal), data curation (equal), formal analysis (equal), investigation (equal), methodology (equal), resources (equal), supervision (equal), writing – original draft (equal), writing – review and editing (equal). **Feng Xu:** conceptualization (equal), data curation (equal), formal analysis (equal), investigation (equal), methodology (equal), resources (equal), supervision (equal), validation (equal). **Gui‐qing Li:** conceptualization (equal), data curation (equal), investigation (equal), methodology (equal), software (equal), validation (equal), visualization (equal). **Jian Chen:** conceptualization (equal), data curation (equal), formal analysis (equal), investigation (equal), resources (equal), supervision (equal), validation (equal). **Wen‐jie Chen:** conceptualization (equal), resources (equal), supervision (equal), validation (equal). **Jiang‐mei Xu:** conceptualization (equal), resources (equal), supervision (equal), validation (equal). **Xiao‐lin Gao:** conceptualization (equal), resources (equal), supervision (equal), validation (equal). **Shen‐ju Liang:** conceptualization (equal), funding acquisition (equal), investigation (equal), methodology (equal), project administration (equal), resources (equal), supervision (equal), validation (equal), writing – original draft (equal), writing – review and editing (equal). **Gui‐lian Xu:** conceptualization (equal), data curation (equal), formal analysis (equal), funding acquisition (equal), investigation (equal), methodology (equal), project administration (equal), resources (equal), supervision (equal), writing – original draft (equal), writing – review and editing (equal).

## Conflicts of Interest

The authors declare no conflicts of interest.

## Data Availability

The data that support the findings of this study are available from the corresponding author upon reasonable request.
